# Editorial: Unraveling the mysteries of aging and the brain: advancements in understanding neurodegenerative diseases and dementia

**DOI:** 10.3389/fgene.2025.1745907

**Published:** 2025-11-24

**Authors:** Victoria Campos-Peña

**Affiliations:** Laboratorio Experimental de Enfermedades Neurodegenerativas, Instituto Nacional de Neurología y Neurocirugía, Manuel Velasco Suárez, Mexico City, Mexico

**Keywords:** Alzheimer’s disease, aging, dementia, amyloid beta, enthorhinal cortex, basal forebrain

The increase in global life expectancy is unquestionably a substantial advancement, indicative of advances in public health, greater access to education, and improved public health practices, nutrition, and quality of life. Nevertheless, population ageing has profound implications for each country’s social and economic structure, as it is linked to an increase in neurodegenerative diseases. These pathologies, in turn, lead to increased demand for healthcare services and long-term care, as well as disruptions in work and family dynamics. It should also be noted that this increase in life expectancy does not guarantee a satisfactory quality of life.

Unravelling the Mysteries of Aging and the Brain: Advancements in Understanding Neurodegenerative Diseases and Dementia is a special multidisciplinary research issue that compiles and proposes new strategies for addressing central nervous system (CNS) diseases and offers a broad overview of current knowledge regarding the molecular and genetic mechanisms underlying neurodegenerative processes. In this regard, the present compendium of manuscripts has been curated with a view to showcasing the most innovative advances, thereby helping to resolve unanswered questions and open new avenues for developing innovative treatment strategies.

As humans age, they experience age-related cognitive decline. The basal forebrain (BF) has complex connections with the hippocampus (Hip) and the medial prefrontal cortex (mPFC) through circuits, and is involved in cognitive functions. However, it is not known with certainty which circuit is most vulnerable during normal ageing.

To address this issue, Sun et al., conducted a quantitative analysis of whole-brain basal forebrain (BF), hippocampus (Hip), and medial prefrontal cortex (mPFC) inputs during normal ageing. They employed a combination of viral tracking and fluorescence micro-optical sectioning tomography (fMOST). The results showed that the input circuits of the diagonal band nucleus (DBN) were particularly vulnerable during normal ageing, especially the vCA3-DBN circuit. Other alterations observed included a weakening of connectivity between subregions of the olfactory areas (OLF), which could be linked to learning and memory (Sun et al.). These results offer a new perspective on future research on the treatment of age-related cognitive decline by providing an anatomical basis for understanding the selective vulnerability of the BF circuit during normal aging.

A hallmark of neurodegenerative diseases is the impairment of learning and memory processes, which can result in dementia. Among the various dementia syndromes, Alzheimer’s disease (AD) is unquestionably the foremost cause of dementia in aging. In the initial stages of the disease, the entorhinal cortex (EC) exhibits significant damage, establishing its importance as a pivotal region in Alzheimer’s disease progression. It has been established that the EC is responsible for the reception of sensory information from cortical regions, as well as the reception of information from olfactory structures. Once integrated, this information is transmitted to different subfields within the hippocampus (dentate gyrus, CA1, CA2, and CA3). Thereafter, it is distributed to the cortex and subcortical regions, including the septum, striatum, amygdala, and thalamus (Karimani et al.). The presence of elevated excitability of the entorhinal cortex (EC) has been linked to the deposition of amyloid beta (Aβ) and the subsequent propagation of tau to neighboring brain regions, thereby facilitating the progression of AD. Accordingly, the modulation of neuronal activity within the CE could facilitate the exploration of novel perspectives for the diagnosis and treatment of Alzheimer’s disease (AD).

Conversely, recent findings have suggested a correlation between vulnerability to infections and Alzheimer’s disease (AD). A study by Rajendrakumar et al., demonstrated an association between the rs6859 polymorphism in the NECTIN2 gene and Alzheimer’s disease. NECTIN2 is a gene that produces a protein integral to the structure of adherens junctions, which are components of cell-to-cell adhesion. This gene is implicated in the response to infections. The observed association between this polymorphism and AD is partially mediated by pTau-181 levels in cerebrospinal fluid (CSF) (Rajendrakumar et al.). Similarly, observational studies have indicated that individuals with dementia who develop sepsis are more likely to die within 28 days. Despite the study’s numerous limitations, the magnetic resonance imaging analysis conducted by Lan et al., demonstrated a causal association between Lewy body dementia and the condition under investigation. In contrast, other forms of dementia showed no evidence of causality (Lan et al.).

Regarding early-onset Alzheimer’s disease (EOAD), Li et al. conducted a proteomic analysis of EOAD brain tissue to elucidate the biological processes and key proteins involved in disease progression. The authors integrated proteomic data from brain tissue of two cohorts of patients with AD and constructed a protein co-expression network. The study identified 2,749 proteins associated with the pathology. Using protein co-expression network analysis, two key proteins were identified and validated. ERBB2IP and LSP1. These proteins may play a fundamental role in the progression of AD, suggesting that they could be potential therapeutic targets for the disease (Li et al.).

Oxidative stress is another characteristic present in several neurodegenerative diseases, such as Alzheimer’s disease. A multitude of studies have indicated its pivotal function in the etiology and progression of AD. A substantial proportion of the oxidative stress evident in the initial stages of AD is associated with neurotoxicity induced by Aβ. Aβ has been demonstrated to be highly toxic to neuronal cells, and it has been posited that neuronal death is attributable to apoptosis and the oxidative effects of this peptide. In their study, Komaki et al. investigated the protective and therapeutic effects of olanzapine (OLZ) in an animal model of Aβ-induced neurotoxicity, utilizing behavioral assessments to evaluate the model’s outcomes. The results obtained demonstrated that treatment with OLZ ameliorated cognitive impairment and diminished anxious behavior in rats that were injected with Aβ. Consequently, the authors posit that OLZ may possess both preventive and therapeutic potential for AS (Komaki et al.).

In conclusion, the articles in this Research Topic provide novel insights into the mechanisms underlying the neurodegenerative process in Alzheimer’s disease. A critical component of this research is to elucidate the brain connections implicated in the neurodegenerative process that culminates in dementia. Furthermore, genetic association studies have identified novel biomarkers that hold promise for facilitating early diagnosis. Conversely, proteomic analyses have facilitated the identification and validation of target proteins that could contribute to the development of new drugs that enhance patients' quality of life, reduce disability, and promote a dignified lifespan.

